# The Agassiz Expedition

**Published:** 1866-06

**Authors:** 


### THE AGASSIZ EXPEDITION.

The Boston *Transcript* contains the following intelligence as to the reception of Professor Agassiz and his party at Rio: "We are glad to learn that Mr. Agassiz and his party arrived safely at Rio de Janeiro on the 22d of April, and were most kindly received. The Emperor sent a boat alongside the Colorado to take the party on shore, and in the evening had a long interview with Mr. Agassiz. The Secretary of the Treasury of Brazil gave orders to have the baggage and instruments of the party passed unopened at the custom house, and every courtesy was extended to the members of the expedition by the officials of the Brazilian government."

